# Speech vs. singing: infants choose happier sounds

**DOI:** 10.3389/fpsyg.2013.00372

**Published:** 2013-06-26

**Authors:** Marieve Corbeil, Sandra E. Trehub, Isabelle Peretz

**Affiliations:** ^1^International Laboratory for Brain, Music and Sound Research, Department of Psychology, Université de MontréalMontréal, QC, Canada; ^2^Music Development Laboratory, Department of Psychology, University of Toronto MississaugaMississauga, ON, Canada

**Keywords:** infants, music, language, singing, speech, emotion, attention

## Abstract

Infants prefer speech to non-vocal sounds and to non-human vocalizations, and they prefer happy-sounding speech to neutral speech. They also exhibit an interest in singing, but there is little knowledge of their relative interest in speech and singing. The present study explored infants' attention to unfamiliar audio samples of speech and singing. In Experiment 1, infants 4–13 months of age were exposed to happy-sounding infant-directed speech vs. hummed lullabies by the same woman. They listened significantly longer to the speech, which had considerably greater acoustic variability and expressiveness, than to the lullabies. In Experiment 2, infants of comparable age who heard the lyrics of a Turkish children's song spoken vs. sung in a joyful/happy manner did not exhibit differential listening. Infants in Experiment 3 heard the happily sung lyrics of the Turkish children's song vs. a version that was spoken in an adult-directed or affectively neutral manner. They listened significantly longer to the sung version. Overall, happy voice quality rather than vocal mode (speech or singing) was the principal contributor to infant attention, regardless of age.

## Introduction

There is considerable debate about similarities and differences in the processing of language and music (e.g., Pinker, 1997; Patel, 2008; Jackendoff, 2009; Peretz, 2009). Because the greatest differences arise from the presence of propositional meaning in language but not in music, comparisons in the early pre-verbal period are of particular interest (Trehub et al., 1993; Chen-Hafteck, 1997; McMullen and Saffran, 2004; Brandt et al., 2012), notably when both modes of parental communication are used to regulate infant attention and affect (Fernald, 1992; Papoušek, 1994; Kitamura and Burnham, 2003; Trehub et al., 2010). To date, however, the only study comparing young infants' behavioral responsiveness to speech and singing (Nakata and Trehub, 2004) used audiovisual stimuli, obscuring the relative contributions of auditory and visual expressiveness to infants' greater engagement with maternal music. Another study found no difference in newborns' neural responses to happy-sounding speech and singing (Sambeth et al., 2008). The present investigation examined infants' attentiveness to speech and singing on the basis of auditory cues alone.

Whereas verbal aspects of speech convey propositional meaning, non-verbal or prosodic aspects such as intonation and rhythm convey the speaker's affective intent and emotional state (Frick, 1985). Mothers across cultures speak and sing to their pre-verbal infants in the course of providing care (Fernald, 1992; Trehub and Trainor, 1998; Dissanayake, 2000; Trehub, 2000). Their manner of speaking or singing to infants (infant-directed or ID) differs dramatically from their manner in other contexts (adult-directed or AD; self-directed or non-ID)(Ferguson, 1964; Jacobson et al., 1983; Fernald and Simon, 1984; Trainor et al., 1997; Trehub et al., 1997a,b), with notable variations across cultures (Grieser and Kuhl, 1988; Fernald et al., 1989; Kitamura et al., 2002). In general, ID speech features higher pitch, expanded pitch contours, slower speaking rate, longer vowels, larger dynamic range, and greater rhythmicity and repetition than AD speech (Stern et al., 1982, 1983; Fernald and Simon, 1984; Fernald et al., 1989). These features, especially high pitch, expanded pitch contours, rhythmicity, repetition, and reduced speaking rate, make ID speech sound much more musical than AD speech (Fernald, 1989, 1992). High pitch, expanded pitch contours, and large dynamic range also reflect the heightened affective quality of typical ID speech, which contrasts with the affective restraint of typical AD speech (Trainor et al., 2000). Nevertheless, ID speech is finely tuned to the infant's age and needs, with mothers using relatively more comforting speech for 3-month-olds, more approving speech for 6-month-olds, and more directive speech for 9-month-olds (Kitamura and Burnham, 2003). Approving speech, with its higher pitch and greater pitch range, receives higher ratings of positive affect by adult listeners (Kitamura and Lam, 2009).

Unlike speech, singing is constrained by the prescribed pitch and rhythmic form of the material (i.e., specific songs). Nevertheless, ID versions of singing are also characterized by higher pitch and slower tempo than non-ID versions of the same songs by the same singers (Trainor et al., 1997; Trehub et al., 1997a,b). While repetition is an important aspect of ID speech, it is central to music in general (Kivy, 1993; Trainor and Zatorre, 2008) and to songs for young children in particular (Trehub and Trainor, 1998).

The available evidence indicates that infants find ID singing more engaging than non-ID singing (Trainor, 1996; Masataka, 1999) just as they find ID speech more engaging than AD speech (Fernald, 1985; Werker and McLeod, 1989; Pegg et al., 1992). One possible source of infants' enhanced engagement is the heightened positive expressiveness of typical ID speech and singing (Trainor et al., 2000; Trehub et al., 2010; Nakata and Trehub, 2011). In fact, infants exhibit preferential listening to speech that sounds happy rather than sad or inexpressive regardless of the intended audience (Kitamura and Burnham, 1998; Singh et al., 2002). For example, infants listen longer to happy AD speech than to affectively neutral ID speech even when the latter is higher in pitch (Singh et al., 2002). Note, however, that happy ID vocalizations are closer to AD vocalizations described as *high-arousal joy/happiness* or elation than to *low-arousal joy/happiness* (Banse and Scherer, 1996; Bänziger and Scherer, 2005). Infants also exhibit more positive affect to ID expressions of approval than to disapproval or prohibition even when the utterances are low-pass filtered (Papoušek et al., 1990) or presented in an unfamiliar language (Fernald, 1993). The general consensus is that positive vocal emotion, especially the high arousal variety, makes a substantial contribution to infants' interest in ID speech. Nevertheless, one cannot rule out alternative explanations such as the attention-getting potential of expanded pitch and dynamic range and the attention-holding potential of repetition. When these acoustic factors are controlled, however, infants exhibit preferences for the happier speech version (Kitamura and Burnham, 1998; Singh et al., 2002), suggesting that these acoustic features make secondary contributions to infant preferences. Infants' interest is also affected by their age and corresponding needs. For example, 3-month-old infants exhibit greater attention to comforting than to approving ID speech (Kitamura and Lam, 2009).

The influence of ID pitch contours is seen in infants' preferential listening for sine-wave replicas of ID speech that preserve the pitch contours (and timing) with uniform amplitude over those that preserve the timing and amplitude with unvarying pitch (Fernald and Kuhl, 1987). Despite the fact that infants display greater positive affect to approving than to disapproving ID utterances, they listener longer to the former only if they exhibit greater F0 modulation (Fernald, 1993). Interestingly, pitch modulation also makes important contributions to the differentiation of emotions in music and in AD speech (Scherer, 1986, 1995; Laukka et al., 2005). Across cultures, happy-sounding speech and music feature high mean pitch, large pitch variability, relatively high mean amplitude, and rapid rate or tempo (Juslin and Laukka, 2003). Smiling elevates pitch and increases amplitude by altering the mouth opening and shape of the vocal tract, contributing to the vocal qualities associated with happiness (Tartter, 1980). Tender speech and music, by contrast, have lower mean pitch, pitch variability, mean amplitude, and slower rate or tempo than happy speech and music (Juslin and Laukka, 2003).

Perhaps the two classes of songs for infants, lullabies and play songs, are caregivers' expressions of tenderness and happiness, respectively, as well as tools for soothing or amusing infants. In line with their soothing function, lullabies feature very slow tempo, low pitch, falling pitch contours, limited amplitude variation, and soothing tone of voice (Unyk et al., 1992; Trehub et al., 1993; Trehub and Trainor, 1998), properties that are shared with soothing ID speech (Papoušek and Papoušek, 1981; Fernald, 1989). Lullabies are also soothing to adult listeners, so it is not surprising that they are used, at times, as laments (Trehub and Prince, 2010) and in palliative care (O'Callaghan, 2008). Although play songs are commonly sung to Western infants, they are not universal, as lullabies are (Trehub and Trainor, 1998).

Maternal speech melodies are considered central to the expression of maternal affect and the regulation of infant attention and arousal (Fernald, 1992; Papoušek, 1994). Is it possible that musical melodies would be equally effective or even more effective in regulating infant attention and arousal? The melodies or pitch contours of expressive speech differ from those in music (Zatorre and Baum, 2012). In music, pitches are discrete and sustained, and steps from one pitch level to another are generally small, most commonly, one or two semitones, with larger pitch jumps being much less frequent (Vos and Troost, 1989). By contrast, pitches in speech glide continuously over a larger range (Patel et al., 1998), which is even larger in ID speech (Ferguson, 1964; Stern et al., 1982, 1983; Fernald and Simon, 1984). Moreover, pitches have precise targets in music but not in speech (Zatorre and Baum, 2012).

If the expanded pitch and dynamic range of ID speech underlies infants' greater attention to ID than to AD speech (e.g., Fernald, 1993), then infants could show more interest in ID speech than ID singing. If rhythmicity and predictability are relevant (e.g., McRoberts et al., 2009), then infants might exhibit more attention to ID singing than to ID speech. If positive emotion is the critical feature (Kitamura and Burnham, 1998; Singh et al., 2002), then infants could show greater interest in the stimulus expressing more positive affect regardless of whether it is speech or music. For adults, music generates a range of positive emotions from tranquillity and tenderness to joy and euphoria (Blood and Zatorre, 2001; Menon and Levitin, 2005; Zentner et al., 2008; Salimpoor et al., 2011). Some scholars contend that the expression of emotion by some form of music (e.g., protomusic) preceded language (Darwin, 1871; Mithen, 2005). Others regard speech, even at present, as a type of music, especially when considered in developmental perspective (Brandt et al., 2012). If the status of speech is privileged, as some contend (Vouloumanos and Werker, 2004, 2007; Shultz and Vouloumanos, 2010; Vouloumanos et al., 2010), then ID speech would be favored over forms of singing that exclude speech. Obviously, the aforementioned factors are not independent. Nevertheless, comparisons of infants' responsiveness to speech and music are a first step toward the long-range goal of identifying the acoustic features that attract and hold infants' attention. Such features may differ for infants of different ages, as reflected in age-related changes in listening biases for ID speech with comforting, approving, or directive tones of voice (Kitamura and Lam, 2009) and for regular or slowed ID speech (Panneton et al., 2006).

It is difficult to assess infants' degree of engagement with music and even more difficult to ascertain their aesthetic preferences. Instead of overt affective responses to music, infants commonly exhibit interest or attention, sometimes accompanied by reduced motor activity (Nakata and Trehub, 2004). The usual assumption is that longer listening to one of two auditory stimuli reflects preference or greater liking for that stimulus (e.g., Fernald and Kuhl, 1987; Trainor, 1996; Vouloumanos and Werker, 2004). In general, such “preferences” are assessed with the head-turn preference procedure, which is used with infants as young as 2 or 3 months of age (e.g., Trainor et al., 2002; Shultz and Vouloumanos, 2010). The procedure involves pairing one auditory stimulus with a visual display and a contrasting auditory stimulus with the same visual display, at the same or different locations, on a series of trials. Infants control the procedure in the sense that looking away from the visual stimulus terminates the auditory stimulus. In other words, they can *choose* to listen to one stimulus longer than another. The interpretation of longer or shorter listening times as positive or negative aesthetic evaluations is questionable in the absence of positive or negative affective displays (Trehub, 2012). At times, infants listen longer to familiar stimuli and, at other times, to novel stimuli (e.g., Rose et al., 1982; Volkova et al., 2006; Soley and Hannon, 2010). Even when infants show positive affect to one auditory stimulus and negative or neutral affect to another, their listening times to the stimuli may not differ (Fernald, 1993). Unquestionably, looking or listening times indicate infants' listening choice or relative attention to the stimuli, but the factors that contribute to such attention are unclear. Some listening biases may be innate, arising from the salience of biologically significant stimuli (e.g., human vocal sounds) or biologically significant parameters of sound (e.g., loud or unexpected). Other listening biases may arise from acquired salience, as in preferential responding to the sound of one's name (Mandel et al., 1995) or to a stimulus heard previously (Zajonc, 2001). Attention biases, regardless of their origin, are likely to facilitate learning (Vouloumanos and Werker, 2004).

In addition to the well-documented listening bias for ID over AD speech, there are reported biases for vocal over non-vocal sounds (Colombo and Bundy, 1981; Vouloumanos and Werker, 2004, 2007), speech over non-human vocalizations, (Vouloumanos et al., 2010), speech over human non-speech vocalizations (Shultz and Vouloumanos, 2010), musical consonance over dissonance (Trainor and Heinmiller, 1998; Zentner and Kagan, 1998), and familiar over unfamiliar musical meters (Soley and Hannon, 2010). Infants also exhibit considerable interest in vocal music (Glenn et al., 1981), but their exposure to music is much more limited than their exposure to speech (Eckerdal and Merker, 2009). To date, however, there has been little exploration of infants' relative interest in speech and singing. In the single study that addressed this question directly (Nakata and Trehub, 2004), 6-month-olds infants watched audio-visual recordings of their mother singing or speaking from an earlier interaction. Infants showed more intense and more sustained interest in singing than in speech episodes, as reflected in greater visual fixation coupled with reduced body movement. Infants' heightened interest in these maternal singing episodes could stem from mothers' propensity to smile more when singing than when talking to infants (Plantinga et al., 2011). In the present study, we used the head-turn preference procedure to assess infants' interest in speech and singing with unfamiliar materials and voices. As noted above, the procedure provides information about infants' listening choices or relative attention rather than their aesthetic preferences.

In line with age-related changes in infants' attention to the affective tone of ID speech (Kitamura and Lam, 2009), developmental changes might be evident in infants' responsiveness to ID speech and song. Accordingly, infants in the present research, who were 4–13 months of age, were divided into three age groups to explore the possibility of comparable age-related changes. In Experiment 1, infants were exposed to ID or happy-sounding speech syllables and soothing hummed lullabies produced by the same woman. The principal question concerned the relative efficacy of soothing hummed song and happy ID speech for attracting and maintaining infants' attention. In other words, is vocal music compelling for infants, as it is for adults, even in the absence of speech or properties associated with heightened arousal? If infants listened longer to hummed lullabies than to simple ID speech, it would challenge the prevailing view that infants have an innate or early developing preference for speech over any other auditory stimulus (Vouloumanos and Werker, 2004, 2007; Shultz and Vouloumanos, 2010; Vouloumanos et al., 2010). Experiments 2 and 3 narrowed the differences between speech and singing stimuli by comparing the same verbal materials that were spoken or sung with comparable or contrasting affective intentions. Specifically, infants in Experiment 2 heard sung vs. spoken renditions of the lyrics of a Turkish children's song, both in an ID/joyful manner. Infants in Experiment 3 heard the ID children's song vs. a spoken version of the lyrics in an AD or affectively neutral manner.

All of the stimuli in the present study were portrayed or acted rather than being recorded during actual interactions with infants and adults. Early research on infants' responsiveness to ID and AD speech (e.g., Fernald, 1985) used recordings of women's interactions with their infant and with an adult experimenter. Such stimuli differed dramatically in content as well as expressiveness, making it difficult to identify the factors contributing to infants' responsiveness. Later research used portrayals of ID and AD speech (e.g., Singh et al., 2002; Kitamura and Lam, 2009) so that the content could be carefully controlled across speech registers. When studying infants' responsiveness to ID and non-ID singing (e.g., Trainor, 1996; Masataka, 1999), it is possible to use recordings of mothers singing the same song in the presence or absence of their infant. Comparisons of natural ID speech and singing (e.g., Nakata and Trehub, 2004), however, necessarily differ in content as well as form. Because the features of ID speech and singing have been described extensively (e.g., Ferguson, 1964; Trainor et al., 1997), it is possible to create relatively natural portrayals of those stimuli. For practical as well as ethical reasons, most of the research on vocal emotion (e.g., Scherer, 1986, 1995; Juslin and Laukka, 2003) has used portrayals of various emotions rather than emotional expressions produced in natural contexts.

## Experiment 1

The goal of the present experiment was to examine the possibility that infants might be more responsive to vocal music than to happy ID speech even for vocal music lacking the acoustic features (e.g., highly variable pitch and dynamics) and expressive intentions (high-arousal happiness) that have been linked to infant preferences for ID speech (e.g., Fernald, 1985; Singh et al., 2002). By using hummed songs, it was possible to generate vocal music without speech. Humming, usually with closed mouth, can be used to generate melodies with sustained nasal sounds that have low spectral amplitude (Kent et al., 2002). Because humming constrains amplitude modulation, it provides reduced scope for expressing high-arousal emotions. There are speculations, however, that humming played an important role in early hominid evolution, functioning like contact calls in other species (Jordania, 2010). At present, humming may be the most common type of informal, solitary singing.

We considered lullabies the musical genre of choice because of their suitability for humming, their universal use in caregiving (Trehub and Trainor, 1998), and their stark contrast with happy ID speech in acoustic features and affective intentions. As noted, lullabies transmit positive affective qualities such as tranquillity and tenderness both in their musical features and vocal tone. The ID speech stimuli approximated those used in previous research on infants' listening biases for speech (Vouloumanos and Werker, 2004, 2007). They consisted of nonsense syllables with typical exaggerated pitch contours and happy voice quality. For adults, it is likely that the lullabies, although unfamiliar, would have high aesthetic appeal, while the repetitive, high-pitched nonsense syllables would sound boring or worse. Nevertheless, the speech combined the exaggerated pitch contours and joyful expressiveness that have been linked to infant preferences in contemporary urban cultures (Fernald and Kuhl, 1987; Kitamura and Burnham, 1998; Singh et al., 2002). If infants share adults' aesthetic appraisals or favor universal forms, they would listen longer to the hummed versions of traditional lullabies. On the basis of previous research with Western infants, however, one might expect them to listen longer to the arousing and joyfully rendered speech.

### Method

#### Participants

The sample consisted of 50 healthy, full-term infants who were 4.3–13.1 months of age (*M* = 8.6 months, *SD* = 2.6) divided into 3 age groups: 4–6 months (*M* = 5.5, *SD* = 0.48; *n* = 16), 7–9 months (*M* = 8.6, *SD* = 0.87; *n* = 16) and 10–13 months (*M* = 11.5, *SD* = 0.74; *n* = 18). No infant had a family history of hearing loss or personal history of ear infections, and all were free of colds or ear infections on the day of testing. An additional five infants failed to complete the test session because of fussiness. This experiment and others in this report were approved by the Arts and Sciences ethics committee of the University of Montreal, and written informed consent was obtained from all participating parents.

#### Stimuli

The speech stimulus, which was comparable to that used by Vouloumanos and Werker (2004) except for a different speaker, consisted of 12 variations of each of two nonsense syllables (*lif* and *neem*) spoken with ID prosody. Varied repetitions of each syllable had rising, falling, and rising-falling (i.e., bell-shaped) pitch contours. There were two versions of the syllabic sequence, differing only in the order of elements. Each sequence consisted of a semi-random ordering of syllables, with the constraint that any four consecutive syllables contained two instances each of *li*f and *neem*. Syllables were separated by silent inter-stimulus intervals (ISIs) of 300–500 ms, and the order of ISIs was randomly distributed, with a mean of 450 ms, as in Vouloumanos and Werker (2004). Each sequence was approximately 20 s in duration, and was repeated for an overall duration of 40 s. The music stimulus consisted of a hummed version of a lullaby. There were two traditional lullabies, one Chilean (in duple meter, AA form) and one German (in triple meter, AB form), each approximately 40 s in duration and each assigned to half of the infants. Hummed and spoken stimuli were produced by a native speaker of English who had considerable music training, singing experience, and experience with children. She was instructed to produce the nonsense syllables in a lively ID manner and to hum the melodies as if lulling an infant to sleep. She listened to many samples of ID speech and singing beforehand (including the Vouloumanos and Werker syllables) and used pictures of infants to help induce the appropriate mood for her speaking or lulling. Sample stimuli are presented in Supplementary Materials.

**Figure 1:**
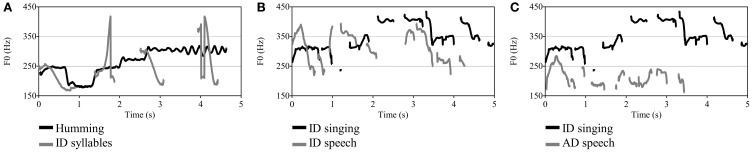
**Fundamental frequency (F0) contours of 5-s excerpts from each sound type. (A)** hummed lullaby (Chilean) and syllable sequence, **(B)** ID sung and spoken lyrics of Turkish play song, **(C)** ID sung and AD spoken lyrics of Turkish play song.

Acoustic features of the stimuli, which were measured with Praat software (Boersma and Weenink, 2010), are shown in Table 1. Because pitch extraction software is prone to octave errors, it is common to manually specify a minimum and maximum fundamental frequency (F0 in Hz) or to use a formula for setting the F0 range of each sound such as that suggested by De Looze and Hirst (2008): floor = q25 × 0.75; ceiling = q75 × 1.5. We used this formula for acoustic analyses in the present study. Mean F0 was higher for singing (*M* = 280.2 Hz) than for speech (*M* = 244.2 Hz, difference of 2.46 semitones), but speech was more variable in F0, amplitude, and timing. The standard deviation (*SD*) of F0, a measure of pitch variability, was 3.81 and 3.40 semitones for speech and singing, respectively. As can be seen in Figure 1, which depicts the F0 contours, changes in pitch were larger and more abrupt for the speech than for the humming stimuli. Amplitude variation (*SD*), measured in the voiced portions of each sound, was 9.31 dB for speech and 4.46 dB for singing. The timing of the syllables was varied deliberately as in Vouloumanos and Werker (2004).

**Table 1:** **Acoustic features of stimuli**.

	**Stimuli**				
	**Experiment 1**		**Experiments 2 and 3**		
	**Humming**	**Syllables**	**ID singing**	**ID speech**	**AD speech**
F0 mean (Hz)	280.20	244.23	351.14	312.28	210.24
F0 SD (semitones)	3.40	3.81	2.34	3.86	2.30
F0 range (semitones)	14.77	18.22	11.41	17.64	11.33
Overall duration	–	–	26.8	24.6	19.02

#### Apparatus

Testing was conducted in a sound-attenuating booth (IAC) 4 by 4 m in size. Infants were seated on their parent's lap facing a central computer monitor at a distance of 127 cm, with two identical monitors to the right and left side of the central monitor and at a distance of 152 cm from infants. Parents wore earphones (ER-4 MicroPro with reusable ER-4S eartips) with an approximate attenuation of 35 dB and earmuffs (Peltor H10A, Optime 105) with an approximate attenuation of 30 dB. They heard continuous music through the headphones to mask the sounds presented to infants. The walls and table for the monitors were covered with black cloth to reduce visual distraction and optimize attention to the target stimuli. A camera immediately above the central screen provided a continuous record of infant visual behavior on a monitor outside the booth. Two loudspeakers (Genelec 8040A) located behind the lateral monitors transmitted the sounds at a comfortable listening level, approximately 60–65 dB (A). The procedure was controlled by customized software on a computer (Mac Pro 8 cores) located outside the booth.

#### Procedure

The head-turn preference procedure (Kelmer Nelson et al., 1995) was used. Infants remained seated on their parent's lap throughout the procedure, and parents were asked to minimize their own movement. Infants were randomly assigned to one of the two speech sequences and one of the two hummed lullabies. The speech and singing stimuli were presented on 10 alternating trials, with order of stimuli (speech or singing first) and side of presentation (left or right) counterbalanced across infants. On each trial, the infant's attention was attracted to one monitor by a flashing red square. As soon as the infant looked at that monitor, one sound stimulus was presented together with a visual animation of a carousel. When the infant looked away from the monitor for more than 2 s, the visual and sound stimuli were terminated. The infant's attention was then attracted to the other monitor. Looking at that monitor initiated the same visual stimulus but the contrasting auditory stimulus, which continued until the infant looked away for 2 s. On each trial, the stimulus was always presented from the beginning (i.e., beginning of the lullaby or syllable sequence). The experimenter outside the booth, who had no access to sound (auditory stimuli or infant vocalization) and no information about test conditions, observed the infant's behavior on the external monitor and continuously recorded looking toward or away from each monitor in the booth by means of key codes on a computer keyboard. Looking times during the presentation of each stimulus type were computed automatically. Typically, infants completed the procedure in approximately 5 min.

### Results

**Figure 2:**
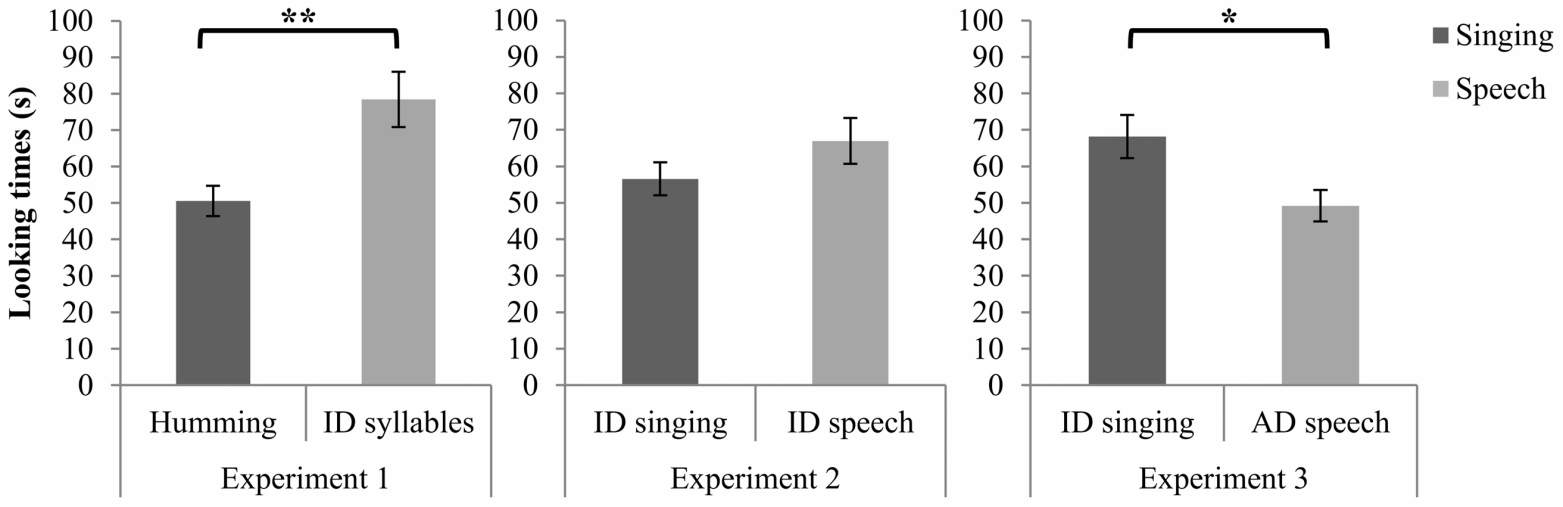
**Cumulative looking time in seconds (s) for singing and speech**. Error bars are standard errors (^**^p < 0.01; ^*^p < 0.05).

Infants often look disproportionately long on the initial trial of a novel stimulus, so it is common to exclude the first two trials (i.e., initial exposure of each stimulus) from data analysis (e.g., Vouloumanos and Werker, 2004; Volkova et al., 2006), a procedure followed here. These initial trials can be considered familiarization rather than test trials. Missing values from infants (4 incomplete trials: 1–2 trials from 3 infants) were replaced with the multiple imputation method (Graham, 2012) implemented with NORM software (Schafer, 1999). Substitution or omission of those values yielded similar results. A preliminary analysis of variance revealed that the effect of age (4–6, 7–9, 10–13 months) on looking time was not significant. Age, considered as a continuous variable in a regression analysis, also made no contribution to looking time. Consequently, age was excluded from the main analysis. A paired sample *t*-test on cumulative looking time across the four trials with each stimulus revealed a significant difference between speech and singing [*t*_(49)_ = 3.35, *p* < 0.01, two tailed]. Infants looked longer during the syllable sequences (*M* = 77.93 s, *SD* = 53.98 s) than during the hummed lullabies (*M* = 50.14 s, *SD* = 29.58) (see Figure 2). A binomial test revealed that of the 50 infants in the sample, 36 (72.0 %) had longer looking times for speech, *z* = 3.11, *p* < 0.01.

### Discussion

Infants exhibited greater attention to the ID speech syllables than to the hummed lullabies despite the greater coherence and continuity of the lullabies. Although our findings are consistent with the speech bias that has been proposed for young infants (Vouloumanos and Werker, 2004, 2007), there are a number of alternative interpretations. The stimuli contrasted in other respects than the presence or absence of speech or syllabic content. For one thing, the speech was considerably more variable than the humming in pitch and amplitude. Typical ID speech has much more continuity than the present sequence of disconnected syllables, each of which had the properties of stressed syllables. Moreover, each of the syllables had the exaggerated pitch contours that are considered critical in capturing infant attention (e.g., Fernald and Kuhl, 1987), and these contours were highly variable. The speech stimuli also had bursts of very high-pitched sound at irregular time intervals (see Figure 1), which could have functioned as salient alerting signals. Hummed speech produces less neural activation than natural speech (Perani et al., 2011), so one would expect hummed music to produce less cortical activation than other types of vocal music.

The affective qualities of the stimuli also differed dramatically, with the speech having the properties of high-arousal happiness or joy and the hummed lullabies being tranquil and soothing. Joyful or happy speech reliably attracts and maintains the attention of Western infants (Singh et al., 2002), and joyful music may do likewise. In contrast to Western mothers, who engage in lively vocal and non-vocal interactions with their infants, mothers in many others cultures interact in ways that are primarily soothing rather than arousing (Toda et al., 1990; Trehub and Schellenberg, 1995; Trehub and Trainor, 1998). It is possible that infants who are accustomed to soothing vocal interactions would distribute their attention differently from the infant participants in the present experiment. Nevertheless, the youngest infants in the present study, who might experience more soothing interactions than the older infants (Kitamura and Burnham, 2003), responded no differently than the older infants.

Finally, the stimuli in the present study were atypical in a number of respects. The speech stimulus had the usual exaggerated pitch contours and happy affect of Western mothers in the unusual context of two single, non-contiguous syllables that repeated with variable renditions (following Vouloumanos and Werker, 2004, 2007). In other words, it was dramatically different from conventional ID speech. Although lullabies, sung or hummed, are common in non-Western cultures, they are used infrequently in Western cultures (Trehub and Trainor, 1998). If Western infants are exposed to lullabies, such exposure typically occurs when they are sleepy or distressed rather than awake or alert. For those reasons, we used more conventional stimulus materials in subsequent experiments, namely the lyrics of foreign children's songs that were spoken or sung.

## Experiment 2

The goal of the present experiment was to ascertain the relative efficacy of speech and singing for maintaining infant attention when verbal or syllabic content and affective intentions are similar across vocal modes. Infants were presented with a sung and spoken version of an unfamiliar Turkish play song, both produced in an ID or joyful manner. The same lyrics ensured comparable phoneme sequences despite their different realization in speech and singing. Although the overall affective intentions were joyful in both cases, the means of achieving those intentions differ in speech and singing, with unknown consequences.

In research with ID and AD speech, the stimuli are often drawn from natural interactions with infants and adults (e.g., Kitamura and Burnham, 1998) so that verbal content and speaking style differ. At other times, actors portray ID and AD speech with the same verbal content (e.g., Singh et al., 2002). No previous study used the texts of play songs, which include words and nonsense syllables that are distinctive and memorable as well as alliteration, assonance, and rhyme. As a result, the spoken ID version was closer to a spoken nursery rhyme than to conventional ID speech, reducing many of the usual differences between spoken and sung material for infants. Differences between speech and singing still remained, however, with speech being more variable in its pitch patterns and amplitude and also lacking the steady beat of music. If the expanded pitch range and greater pitch variability of speech drive infant attention (e.g., Fernald and Kuhl, 1987; Fernald, 1992), then infants could be expected to attend longer to the spoken lyrics. If happy affect is primarily responsible for infants' listening choices, as is the case for speech style (Singh et al., 2002), then infants might respond no differently to happy ID speech and singing with comparable verbal content.

### Method

#### Participants

The sample included 48 healthy full-term infants who were 4.2–12.4 months of age (*M* = 8.3 months, *SD* = 2.3), with the same inclusion criteria as Experiment 1, and the same age groups: 4–6 months (*M* = 5.7, *SD* = 0.9; *n* = 16), 7–9 months (*M* = 8.5, *SD* = 0.8; *n* = 16), and 10–12 months (*M* = 10.8, *SD* = 0.8; *n* = 16). An additional 6 infants were excluded from the final sample because of experimenter error (*n* = 2) or failure to complete the test session (*n* = 4).

#### Stimuli

Stimuli consisted of unfamiliar foreign lyrics (Turkish) of a play song (duple meter, AABAA form) that were spoken or sung. The performer was a native Turkish speaker and trained singer who had considerable experience with children. She listened to many samples of ID speech and singing and was instructed to speak and sing as if doing so for an infant. Stimuli are available in Supplementary Materials. Acoustic features of the sounds, as analyzed by Praat software (Boersma and Weenink, 2010) with pitch range settings following Experiment 1, are shown in Table 1. Sung versions were slightly longer than spoken versions, 26.8 s vs. 24.6 s. Mean pitch level was 2.3 semitones higher for sung (*M* = 351.14 Hz) than spoken versions (*M* = 312.28 Hz), but spoken versions had considerably greater pitch range (17.64 vs. 11.41 semitones) and pitch variability (*SD*s of 3.86 and 2.34 semitones, respectively). The mean pitch of the sung lyrics was substantially higher for the highly trained Turkish singer than for mothers' ID singing of play songs (253.6 Hz) (Trainor et al., 1997), but the pitch level of the spoken lyrics was comparable to that of mothers' ID speech (Fernald et al., 1989). As can be seen in Figure 1, however, there was more overlap of the ID speech and singing contours than was the case for Experiment 1.

#### Apparatus and procedure

The apparatus and procedure were identical to Experiment 1.

### Results

As in Experiment 1, a preliminary ANOVA revealed no effect of age on looking time, so age was excluded from the main analysis. A paired sample *t*-test on cumulative looking time across four trials with each stimulus (initial two trials omitted, as in Experiment 1) revealed no difference between speech (*M* = 66.97 s, *SD* = 43.24 s) and singing (*M* = 56.58 s, *SD* = 31.57 s) [*t*_(47)_ = 1.30, *p* = 0.199, two tailed] (see Figure 2).

### Discussion

Infants' attention did not differ for spoken and sung versions of a Turkish play song performed in an ID manner. The absence of differential attention, even in the presence of greater pitch and duration variability of the spoken versions (i.e., lively and rhythmic ID speech), implies that such acoustic variability, in itself, cannot account for the attention differences in Experiment 1 or in previous research (Nakata and Trehub, 2004). The findings raise the possibility that happy vocal affect, which characterized the spoken and sung versions, is primarily responsible for infants' engagement. Affective voice quality may be transmitted, in part, by the acoustic features that were measured but it is also transmitted by vocal timbre (i.e., tone of voice), which is not readily amenable to quantification. Issues of affective intent were addressed in the subsequent experiment.

## Experiment 3

In the present experiment, we altered the affective intent of the spoken lyrics of Experiment 2 for comparison with the ID sung lyrics. Infants were exposed to the ID sung version from Experiment 2 and a spoken version in a non-ID style with neutral affect. If infants' attention is driven primarily by the joyful or happy quality of adult vocalizations, then they should exhibit greater attention to the sung versions than to the spoken versions. Just as infants are more engaged by happy speech than by neutral speech regardless of the ID or AD register (Kitamura and Burnham, 1998; Singh et al., 2002), we expected them to be more engaged by happy than by neutral vocal material regardless of whether it was spoken or sung.

### Method

#### Participants

The sample included 48 healthy, full-term infants who were 4.7–12.5 months of age (*M* = 8.3 months, *SD* = 2.5). Inclusion criteria were comparable to Experiment 1, as were the age groups: 4–6 months (*M* = 5.7, *SD* = 0.7; *n* = 16), 7–9 months (*M* = 8.0, *SD* = 0.9; *n* = 16), and 10–12 months (*M* = 11.3, *SD* = 0.8, *n* = 16). An additional five infants were excluded from the final sample because of failure to complete the test session (*n* = 4) or parents' interaction with infants during the test session (*n* = 1).

#### Stimuli

Stimuli consisted of the same sung lyrics of the Turkish play song used in Experiment 2, which was unfamiliar to infants or mothers, and an affectively neutral version of the spoken lyrics. The lyrics were spoken by the same native Turkish speaker from Experiment 2, who was instructed to speak with neutral affective tone as if communicating with an adult. Stimuli are available in Supplementary Materials. Acoustic features of the sounds (analyzed by means of Praat software) are shown in Table 1. Pitch range setting followed the procedures described in Experiment 1. The **s**ung version was substantially longer (26.8 s) than the spoken version (19.02 s), reflecting the slow pace of singing relative to ordinary speech. Mean pitch level for the sung and spoken versions was 350.14 and 210.24 Hz, respectively, corresponding to a difference of 8.9 semitones. F0 variability (SD) for the spoken and sung lyrics was similar at 2.30 and 2.34 semitones, respectively, as was the pitch range (i.e., difference between minimum and maximum pitch) of 11.33 and 11.41 semitones, respectively (see Figure 1). In short, the singing and speech stimuli differed substantially in pitch level, rate, and vocal tone (happy vs. neutral) but were comparable in pitch variability and pitch range.

#### Apparatus and procedure

The apparatus and procedure were identical to Experiment 1.

### Results

Missing values for one infant on the final trial were handled by the multiple imputation method (Graham, 2012), as in Experiment 1. Data from one outlier (>3 *SD* from the mean) were excluded from the data set. Inclusion of the outlier and omission of the missing trial did not alter the results. A preliminary ANOVA revealed no effect of age on looking time, so age was excluded from the main analysis. A paired sample *t*-test on cumulative looking time across the four trials for each stimulus type revealed a significant difference between speech and singing [*t*_(46)_ = 2.34, *p* < 0.05, two tailed]. Infants looked longer in the context of singing (*M* = 68.17 s, *SD* = 40.41 s) than in the context of neutral speech (*M* = 49.20 s, *SD* = 29.45) (see Figure 1). A binomial test revealed that, of the 47 infants in the sample, 34 (72.3 %) looked longer during the presentation of singing, *z* = 3.016, *p* < 0.01.

### Discussion

As predicted, infants exhibited greater attention during the presentation of the happy ID singing than during the neutral AD speech. Despite identical lyrics, similar pitch range (but different pitch register), and similar pitch variability of the sung and spoken versions, singing maintained infants' attention more effectively than did speech. The findings are consistent with a critical role for positive vocal affect, specifically happy or joyful vocalizations. An alternative explanation is that infants responded on the basis of pitch register, with the higher register of ID singing attracting their attention more effectively than the lower register of AD speech (see Figure 1). In speech contexts, however, happy vocal affect makes a greater contribution to infant attention than pitch register does (Kitamura and Burnham, 1998; Singh et al., 2002).

## General discussion

The purpose of the present study was to ascertain infants' relative interest in singing and speech. In Experiment 1, infants showed greater attention to happy ID versions of a series of unconnected nonsense syllables than to soothing hummed lullabies. The soothing humming proved to be no match for the effusively spoken syllables, which combined features of alerting vocalizations and joyful speech as well as high acoustic variability. In general, Western mothers' interactions with infants, whether spoken or sung, are lively and playful, in contrast to the soothing interactions and high levels of body contact that prevail in many non-Western cultures (Morikawa et al., 1988; Fernald, 1992; Trehub and Trainor, 1998). Perhaps infants' listening choices to stimuli such as these would differ in different cultures (e.g., non-Western) and contexts (e.g., when infants are experiencing fatigue or distress).

In Experiment 2, infants heard the lyrics of a Turkish play song that were spoken or sung in a lively, joyful manner. Neither the higher mean pitch of the sung versions nor the greater pitch range and pitch variability of the spoken version resulted in differential infant attention, as they have in previous studies of ID and AD speech (Fernald and Simon, 1984; Fernald and Kuhl, 1987) or ID and non-ID singing (Trainor, 1996; Trainor and Zacharias, 1998). Obviously, the absence of a difference does not provide definitive evidence of equivalent interest in the stimuli, but it is consistent with the notion that infants' listening preferences are influenced primarily by the joyful or happy expressiveness of speech and singing. It is also consistent with newborns' comparable right hemisphere responses to lyrics that are spoken or sung in a happy manner (Sambeth et al., 2008).

In Experiment 3, infants' greater interest in the joyfully sung lyrics than in the neutrally spoken lyrics is in line with high positive affect driving infant attention. The speech stimuli of Experiment 1, the speech and singing stimuli of Experiment 2, and only the singing stimuli of Experiment 3 had features associated with vocal expressions of high-arousal happiness or joy (Banse and Scherer, 1996; Bänziger and Scherer, 2005). Taken together, the results of the three experiments are consistent with the possibility that features associated with vocal expressions of high-arousal happiness or joy are the principal determinants of infant preferences. Infants' attention to stimuli reflecting high levels of positive affect has been documented in visual (Kuchuk et al., 1986; Serrano et al., 1995) as well as auditory (Papoušek et al., 1990; Fernald, 1993; Kitamura and Burnham, 1998; Singh et al., 2002) contexts.

Although caregivers' expressive intentions are important for regulating infants' attention, other factors such as timing and pitch patterns may play an independent role. Music is much more predictable than speech in its temporal and pitch structure, generating expectations and the fulfillment of those expectations as the music unfolds (Kivy, 1993; Trainor and Zatorre, 2008; Jones, 2010). Such predictability contributes to the appeal of music for mature listeners (Kivy, 1993), and it may do so for infants as well. Maternal sung performances for infants have even greater predictability than other music, with many mothers singing the same songs at the same tempo and pitch level on different occasions (Bergeson and Trehub, 2002). Although maternal speech, with its frequent repetition of phrases and intonation contours, is much more predictable than AD speech, the contours are usually repeated with different verbal content (Bergeson and Trehub, 2002, 2007). The speech in Experiment 1, consisting of variable renditions of two syllables, carried repetition to an extreme from the perspective of adults, but the predictable content in the context of changing pitch contours may have highlighted those contours. The lullabies were also repetitive, as are most lullabies (Unyk et al., 1992), but repetition occurred on a longer timescale than for the monosyllabic speech sounds.

The slow tempo and minimal amplitude variation of the lullabies de-emphasized the typical rhythmic regularity of music. The Turkish play song was more rhythmic than its spoken counterpart in Experiment 2, but the simple, repetitive lyrics sounded more like a nursery rhyme or poetry than conventional ID speech. Poetry blurs many of the distinctions between speech and singing by its inclusion of rhythm, meter, rhyme, alliteration, and assonance (Tillmann and Dowling, 2007; Obermeier et al., 2013), all of which were featured to varying degrees in the ID spoken and sung versions of the play song. In addition to having several repeated and rhyming syllables, the speech stimuli in Experiment 2 also had wider pitch contours than the sung stimuli. Such pitch contours have been linked to infants' listening bias for ID over AD speech (e.g., Fernald and Kuhl, 1987). Expanded pitch contours may compete with timing regularity for gaining and retaining infants' attention. Differences in pace, timing regularity, and rhythmicity between speech and singing were pronounced in Experiment 3 when singing finally prevailed. Naturally, one would expect infants' attention to be influenced by several factors acting together rather than a single factor (Singh et al., 2002), with some features being more salient than others in different situations. The acoustic parameters of the speech stimuli in Experiments 2 and 3 conformed to conventional differences between Western ID and AD registers (e.g., Fernald and Simon, 1984), with the ID speech having substantially higher mean pitch, a pitch range that was over 6 semitones greater, and a speaking rate that was substantially slower than the AD or neutral versions (Ferguson, 1964; Stern et al., 1982, 1983). In fact, the ID version of spoken lyrics, with its heightened pitch and slowed rate (see Table 1), was much closer to the sung version than it was to the neutral or AD spoken version (see Figure 1).

Obviously, speech and singing are not uniform across persons or contexts, and the differences between them narrow or widen in different situations. ID speech capitalizes on dimensions that are central to music, especially pitch and rhythm, which make it sound more musical than non-ID speech (Fernald, 1992; Trainor et al., 2000). Although maternal speech is more acoustically variable than maternal singing (Bergeson and Trehub, 2002), mothers make their speech more accessible to infants by the use of individually distinctive intonation patterns or tunes (Bergeson and Trehub, 2007).

To the adult ear, speech and singing, even ID speech and singing, are distinct classes. For young infants, however, melodious speech and singing may be variations on a theme. Brandt et al. (2012) suggest that speech is a special form of music, at least from the perspective of pre-verbal infants. Before language achieves referential status, infants may hear human vocal sequences as sound play, which is what music is all about (Brandt et al., 2012). Because speech lacks the constraints of music, it can become music-like without losing the essential properties of speech. Not only does ID speech exaggerate the features of conventional speech; it also incorporates some musical features such as sustained vowels and phrase-final lengthening, exaggerating others such as pitch range expansion (e.g., Fernald et al., 1989). The elevated pitch and slow tempo of ID speech are comparable to the pitch and tempo of ID singing and to music in general. Perhaps ID speech would be misjudged as music in cultures in which vocal music incorporates free rhythm and pitch glides (e.g., Clayton, 2000).

The present study provides support for the view that happy vocalizations or those with high positive affect, whether speech or singing, play an important role in regulating infant attention. The happy talk of Experiment 1 elicited greater infant attention than the soothing humming, and the happy singing of Experiment 3 elicited greater attention than the neutral speech. When speech and singing were both happy, as in Experiment 2, there was no difference in infants' attention. Can one conclude that that there would be no difference in infants' attention to happy speech and singing outside as well as inside the laboratory? Not necessarily. In everyday life, ID vocal interactions typically involve a familiar voice (e.g., parent), familiar content (e.g., frequently sung song, familiar phonemes, repeated syllable sequences), familiar face and facial expressions, as well as physical contact or movement, creating many possibilities for differential responsiveness to multimodal speech and singing. In fact, infants are more attentive to happy maternal singing than to happy maternal speech when the material is presented audiovisually (Nakata and Trehub, 2004).

Finally, the present research examined infants' attention in a series of relatively brief trials, providing insight into the potential of the stimuli for *capturing* their attention rather than *maintaining* it for sustained periods of time. In principle, one stimulus might be better for initial attention capture (e.g., unconnected speech syllables rendered in a happy voice) while another could have greater efficacy for maintaining attention or contentment, preventing distress, or alleviating distress (e.g., coherent passages of speech or singing). Visual fixation, the measure used in the present study, provides a limited perspective on attention and engagement, being imperfectly correlated with physiological and neural measures of infant attention (Richards et al., 2010) and with infant facial affect (Fernald, 1993). We know, for example, that infants move rhythmically to rhythmic music but not to ID or AD speech (Zentner and Eerola, 2010) and that intense infant attention to vocal music initially leads to reduced body movement (Nakata and Trehub, 2004). Maternal singing also modulates infant cortisol levels (Shenfield et al., 2003). Future research with a wider variety of stimuli and measures may resolve the unanswered questions about infants' responsiveness to expressive speech and singing.

### Conflict of interest statement

The authors declare that the research was conducted in the absence of any commercial or financial relationships that could be construed as a potential conflict of interest.
